# Ruptured gallbladder as the first presentation of breast cancer

**DOI:** 10.1186/1477-7819-7-50

**Published:** 2009-06-01

**Authors:** M Jones, J Mathew, KE Abdullah, T McCulloch, KL Cheung

**Affiliations:** 1 Professorial Unit of Surgery, City Hospital, Nottingham, UK; 2 Department of Histopathology, City Hospital, Nottingham, UK

## Abstract

**Background:**

Perforation of the gall bladder as a first presentation of breast cancer has not been reported.

**Case presentation:**

Here we present a case of an elderly lady with acute abdomen with evidence of possible perforation of gall bladder on CT scan. Histopathology of the cholecystectomy specimen revealed invasive lobular breast cancer.

Her metastatic breast cancer with right sided primary discovered subsequent to her presentation with acute abdomen is managed successfully with Anastrozole.

**Conclusion:**

We present a rare case of gall bladder perforation from metastatic breast cancer.

## Background

Lobular carcinomas of the breast have higher prevalence of spread to gastrointestinal tract compared to their ductal counterparts [1]. Although breast cancer metastasis to the gall bladder has previously been reported [2-4], metastasis leading to perforation is very rare. We present this rare case of metastatic breast cancer presenting for the first time as ruptured gallbladder.

## Case presentation

An 84-year-old lady was admitted to hospital with a 12-hour history of severe, central abdominal pain and vomiting. Her abdomen was generally tender and reduced breath sounds were noted at the right lung base. Oxygen saturations were 94% on air and all other basic observations were normal. Liver function tests were also normal.

**Figure 1:**
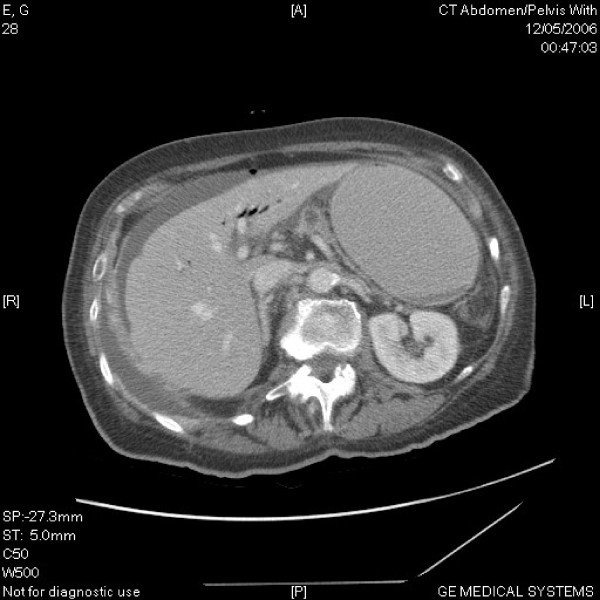
**CT abdomen showing air in the biliary tree and free air in the peritoneum**.

A CT scan demonstrated free air and fluid within the peritoneum, air within the intra-hepatic bile ducts and gallbladder, and a right-sided pleural effusion [Fig 1]. CT scan did not show any obvious evidence of matastatic disease. It was concluded that the gallbladder had perforated and patient was prepared for emergency laparotomy.

She underwent laparotomy, and was found to have a gangrenous, perforated gallbladder containing multiple small gallstones. Cholecystectomy was performed following an attempt of intra-operative cholangiogram which was unsuccessful due to difficulty in cannulating the cystic duct.

**Figure 2:**
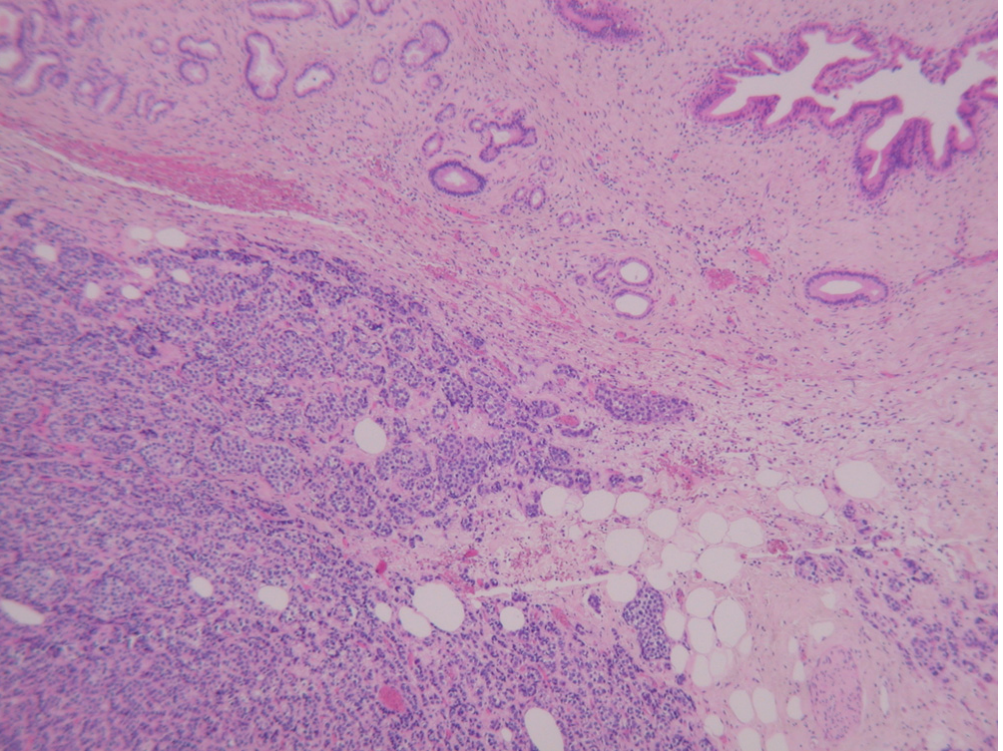
**Metastatic lobular breast carcinoma (bottom left) infiltrating the neck of the gallbladder (top right)**.

Histologically, the lesion appeared to be a metastatic adenocarcinoma [Fig 2]. The gallbladder showed haemorrhagic infarction of the wall, probably caused by an obstructing metastatic carcinoma near the cystic duct. The tumour cells were pleomorphic and were forming glandular structures. Immuno-histochemistry indicated a primary breast tumour as the cells were strongly positive for ER, positive for CK19 and EMA and negative for TTF1, CK20, WT1, CK7, Ca19.9 and Ca125.

A 3.2 × 3.0 cm irregular lump suspicious of cancer was subsequently discovered in the right breast and a 2.9 cm diameter lymph node was palpable in the ipsilateral axilla. The patient had been unaware of these lumps.

Post-operative period was uneventful and she made full recovery. The multidisciplinary team elected to treat her with endocrine therapy and she was therefore started on Anastrozole. She remains asymptomatic and her right sided tumour with axillary metastasis remains stable with Anastrozole even after 34 months of follow-up.

## Discussion

Gall bladder is an uncommon site for metastasis, and in a large series of autopsies with known cancer, gall bladder metastasis was identified in 5.8% of cases [5].

Tumours which commonly metastasise to the gall bladder are malignant melanoma and it occurs in 15% of cases [6,7]. Other less common primary sites leading to secondary metastasis to gall bladder include renal cell cancer, cervical cancer, lung cancer, and breast cancers [8].

Lobular cancers of the breast are well known to metastasise to the gastrointestinal tract compared to ductal cancers, and metastasis to the gallbladder has previously been reported [2-4]. Mechanism behind the affinity for lobular cancers to metastasise to gastrointestinal tract is not well understood. A difference in cell size or shape which favours certain areas of microanatomy that is more contusive to accommodate these cells has been suggested as a possible explanation [1]. It has also been demonstrated that loss of expression of cell to cell adhesion molecule E-cadherin in invasive lobular cancer decreases adhesiveness of cells and could contribute to these differences [9,10].

Bile peritonitis subsequent to metastasis to the gall bladder is extremely rare. The only reported case is an elderly lady with previous history of breast cancer who underwent mastectomy, radiation and chemotherapy many years back, presenting acutely as ruptured gall bladder with associated disseminated metastasis [8].

## Conclusion

Here we report the first case of breast cancer initially presenting as a gallbladder perforation. We postulate that the rupture may be the result of increased pressure in the gallbladder due to obstruction of the cystic duct by metastatic breast carcinoma, which may also explain the difficulty in performing the intra-operative cholangiogram.

## Consent

Written consent was obtained from the patient.

## Competing interests

The authors declare that they have no competing interests.

## Authors' contributions

MJ wrote the report. JM revised and submitted the report for publication. KLC conceived the idea and edited the report. KEA and TMC also helped in editing the report. All authors read and approved the final manuscript.
